# Authors’ Response to Peer Reviews of “Impact of the COVID-19 Pandemic on Latino Families With Alzheimer Disease and Related Dementias: Qualitative Interviews With Family Caregivers and Primary Care Providers”

**DOI:** 10.2196/56439

**Published:** 2024-03-08

**Authors:** Jaime Perales-Puchalt, Jill Peltzer, Monica Fracachan-Cabrera, G Adriana Perez, Mariana Ramírez, K Allen Greiner, Jeffrey Murray Burns

**Affiliations:** 1 Department of Neurology, University of Kansas Alzheimer’s Disease Research Center, Fairway, KS, United States; 2 Department of Family Medicine, University of Kansas Medical Center, Kansas City, KS, United States; 3 School of Nursing, University of Pennsylvania, Philadelphia, PA, United States

**Keywords:** Latino, dementia, caregiving, COVID-19, Alzheimer’s disease, health disparity, qualitative research, transcript analysis, health inequality, minority population, epidemiology, peer support, social services, health care, Alzheimer’s, minority, qualitative, interview, caregiver, primary care, impact, resilience, disparity, outcome, Alzheimer disease, Alzheimer

*This is the authors’ response to peer-review reports for “Impact of the COVID-19 Pandemic on Latino Families With Alzheimer Disease and Related Dementias: Qualitative Interviews With Family Caregivers and Primary Care Providers.”*

## Round 1 Review

Dear Prof. Meinert,

Re: Manuscript ID #42211 entitled “Impact of the COVID-19 Pandemic on Latino Families With Alzheimer Disease and Related Dementias: Qualitative Interviews With Family Caregivers and Primary Care Providers” [1].

Enclosed is a file containing the revised manuscript by Perales-Puchalt et al [1]. Thank you for this opportunity to resubmit the present paper.

We also thank the reviewers very much for their suggestions and comments to our manuscript. We have taken all of them into consideration for producing the new version. In the following sections, we have first presented the reviewers’ comments, followed by our responses.

We hope that these responses and the new manuscript prove satisfactory to you and your reviewers.

We are looking forward to hearing from you. Thank you again for your attention to our work.

Sincerely,

Jaime Perales-Puchalt on behalf of the authors

Fairway, KS; October 12, 2023

### Anonymous [2]

#### General Comments

*This paper describes a qualitative study in which the authors seek to understand the experiences of Latino families managing Alzheimer disease and related dementias (ADRD). The authors interviewed both family caregivers and primary care providers (PCPs). This is a well-written manuscript that focuses on caregiving during COVID-19, a relatively understudied area. The authors note that this study represents secondary analyses of a larger study that focused on improving ADRD care services in primary care across settings.*

#### Specific Comments

##### Major Comments

*1. My key methodological critique is that the authors should follow the COREQ (Consolidated Criteria for Reporting Qualitative Research) recommendations in reporting this study. Since this is a completed study, it is possible that the authors will not meet all the criteria; however, it is still important to know which recommendations were followed and which were not.*

Response: Thank you for the resource! We have edited the manual to follow these guidelines.

*2. My key conceptual critique is that the authors do not provide any rationale as to why family caregivers’ and PCPs’ perspectives are included together. While I certainly understand that these are secondary analyses, the study introduction still needs to justify why these 2 stakeholder groups will provide perspectives that can be synthesized.*

Response: We have clarified in the introduction section that “It is also important to listen to the perspectives of family caregivers and primary care providers (PCPs), who may provide a different point of view and allow triangulation. Given the central role of family in Latino culture [14] and PCPs in the health care system [15], their perspectives can provide privileged insight into the experiences of the person with ADRD, irrespective of their level of cognitive impairment, their family, and their interaction with the health care system.”

*3. Relatedly, the authors should provide a conceptual or theoretical framework that guided this study.*

Response: We added the following to the methods section: “This study was informed by the National Institute on Minority Health and Health Disparities Research Framework, which considers the complex and multifaceted nature of minority health and health disparities [17]. This framework includes different domains of influence (biological, behavioral, physical and built environment, sociocultural environment, and health care system), as well as different levels of influence (individual, interpersonal, community, and societal) within these domains.”

*4. In methods, please clarify whether the main interview was 45-60 minutes and the additional COVID-19–specific questions were excluded from this time. Please also provide the interview guide as an appendix (see COREQ) and include sample questions in the manuscript.*

Response: We have clarified that the 45-60 minutes included the COVID-19 questions.

*5. Please clarify what is meant by “the interviewers emphasized that participants were the experts to reduce power differentials.” How was this accomplished? How did the interviewer ensure that this power differential existed and that it was subsequently reduced?*

Response: We have clarified this sentence, which now says: “The potentially lower theoretical ADRD expertise by family members and PCPs could create a perceived power differential with the interviewer. In fact, some family members and PCPs expressed worry about the interviewer potentially testing their ADRD knowledge. To reduce this perception of power differential, the interviewer emphasized that the interviews would ask about their experiences and that they were the experts in those experiences.”

*6. Consider expanding the description of theme 1 to capture the dimensions of impact noted in the subthemes, for instance, “Both caregivers and PCPs highlighted the physical, psychological and social impacts of the pandemic on patients with ADRD.”*

Response: We have expanded the description of theme 1 to capture the dimensions noted in the subthemes, as suggested by the reviewer.

*7. The second theme does not appear to integrate the PCP and caregiver views as well as theme 1. consider splitting the current theme 2 into 2: theme 2 could be about individual coping and resilience, and theme 3 could be about systems level factors, which would include vaccination acceptance and remote communications.*

Response: We have eliminated the 2 higher levels of themes and included the lower levels. In line with this comment, we have differentiated between individual supports and system supports.

*8. The conclusion discusses death and formal care, which are not highlighted in the themes or results. Anchor the discussion to the results of this study.*

Response: We have carefully linked the discussion with the most important results.

##### Minor Comments

*1. Please review and correct typographical errors; for example, in data analysis, it says “fist author” instead of “first author.”*

Response: We have carefully proofread the manuscript.

*2. The citation #16 is for focus groups, but the authors did 1:1 interviews. Please ensure that this citation is correct.*

Response: Thanks for catching that one. That was an extra citation that was inserted by mistake.

*3. The percentages in Table 1 are not meaningful given the small sample size. Please report only the Ns.*

Response: We have deleted the percentage columns.

### Reviewer FN [3]

#### General Comments

*The authors present a compelling argument for understanding how a doubly vulnerable population in the United States (Latino persons with dementia) experienced the COVID-19 pandemic. To this end, they share the results of a thematic analysis of interviews with primary care providers and caregivers of Latino persons living with dementia. The qualitative analysis could use better explanation and the themes could be more descriptive. Moreover, the many of themes do not capture or explain their relevance to understanding the intersectionality of dementia and Latino lives. My comments below speak to that, as well as other issues. The manuscript has a good foundation of an informative article that showcases the lived experience of this population during a critical time and could be modified to provide formative evidence for improving care, inside and outside of a pandemic.*

#### Specific Comments

##### Major Comments

*1. Consider making subthemes or codes more descriptive and meaningful. Good themes tell you what the story is or what the direction is at least (good or bad). Some of these are readily available (or easily modified) from sentences in the paper already (eg, “the pandemic influence[d] mental and emotional health”; “Social support was critical for reducing social isolation and its sequalae”; caregivers and persons with dementia “lost access to engaging activities during the confinement”; and “Remote communication facilitated social support”). See other qualitative research for examples. There are even good examples in the dementia care literature during the pandemic. For example:*

*Mitchell LL, Horn B, Stabler H, Birkeland RW, Peterson CM, Albers EA, Gaugler JE. Caring for a Relative With Dementia in Long-Term Care During the COVID-19 Pandemic: A Prospective Longitudinal Study. Innov Aging. 2023 Apr 17;7(4):igad034. doi: 10.1093/geroni/igad034. PMID: 37213326; PMCID: PMC10195573.*

*Harding, E., Rossi-Harries, S., Gerritzen, E.V. et al “I felt like I had been put on the shelf and forgotten about” – lasting lessons about the impact of COVID-19 on people affected by rarer dementias. BMC Geriatr 23, 392 (2023). https://doi.org/10.1186.S12877-023-03992-1*

Response: We have taken this comment into account and turned the themes into descriptive statements. We have also further divided them into more specific themes, in line with the ones the reviewer mentioned.

*2. Throughout: The topic is about the Latino ADRD experience, but many themes do not tap into this overlap of the Latino and ADRD experience. It is not made relevant to the ADRD experience or it is not explained how the ADRD context affected it, either in the theme itself (eg, see “poor nutrition” codes) or in the quote used to justify it (eg, see “stress” and “work” codes). A thorough review of the codes and quotes to meet this intersectionality would be beneficial.*

Response: We have thoroughly revised the themes and quotes to highlight the Latino ADRD experience as much as possible. However, while most aspects refer singularly to Latino families with ADRD, some are also experienced by Latino families in general or non-Latino families with ADRD. Including these helps provide information in the discussion section on what impacts are more general to all populations and what things may be particularly salient among Latino individuals.

*3. Facilitators and barriers are not often used as codes on their own but a way to categorize or further delve into aspects of other issues. What was facilitated? What was the barrier blocking? I could see weaving facilitators, barriers, and consequences into the discussions around the other codes, like informal and formal support.*

Response: We have edited our codes, which no longer only state “barriers,” “facilitators,” or “consequences.” Instead, for example, a whole theme includes the barriers to remote communication by Latino families.

*4. Please clarify the methods.*

- *I have not heard of using condensed transcripts before. Why was this done? What was taken out exactly? How were “meaningful bits of text” identified?*
- *It is not clear who was doing the coding at which times. There is the first author as a coder and then 2 additional coders, but the final sentence indicates there were just 2.*
- *Did the first author make all the themes and do all the coding and then the other coder(s) just reviewed it? (Rather than all independently reviewing and coming together to come up with themes and then independently coding and later addressing the coding discrepancies.)*

Response: We have edited this section to further clarify how the coding was done. It now says “JP-P and MF-C independently read the interviews and notes once initially to familiarize themselves with the data, coded the content of the text by identifying codes and themes, and resolved coding disagreements through discussion and consensus.”

*5. Add headers in the text for each subtheme and the codes for better flow and to help readers keep track of what theme or code they are reading about.*

Response: We have added headers and numbers for better flow and to help readers keep track of themes and codes.

*6. For quotes in general:*

- *Consider editing any quote over 2 lines or selecting briefer quotes. Three lines is okay if compelling. If more than 3, it has to be a really good quote. There are 2 very long quotes in “other impacts.”*
- *Provide some context or active linking to the code as lead-in text.*
- *Make sure they are absolutely relevant to the Latino and ADRD experience.*

Response: We have shortened most quotes to make them 1-2 lines long and added more context in the text.

*7. For the discussion:*

- *Consider adding comparisons of findings to non-Latino ADRD COVID-19 experiences to highlight the differences this population experienced and to better showcase why continued attention to this specific population is warranted. See the Mitchell et al and Harding et al papers cited above as potential comparison points.*
- *Why is circular migration being brought up? As written, this does not appear to be ADRD related and was only lightly discussed in the results (though, it was not clear if it is ADRD related in the results either).*
- *“The fact that some PCPs suspected an unknown longer-term impact of the COVID-19 pandemic warrants further longitudinal research into this topic.” While true, it is strange to frame ongoing need to understand this based on participant responses alone. Also, it needs to be related to ADRD.*
- *“Caregivers’ reports on healthcare providers’ confusion between ADRD and COVID-19 infection symptoms warrants research to improve diagnosis and severity assessments of both conditions.” Where did this come from? It feels unsupported from the evidence provided in the current study and an odd place to end the discussion.*

Response: We have focused the discussion on similarities and differences with previous studies on the general population and deleted the discussion on circular migration, diagnosis confusion, and unknown consequences of the pandemic.

*8. For the conclusion:*

- *“This pandemic has revealed many of the barriers that Latino families with ADRD face, and in most cases, this has exacerbated previous barriers. However, with every crisis comes an opportunity for improvement, which will hopefully translate into improved conditions among Latino families with ADRD.” This does not really say anything; be specific regarding the barriers and what could be improved. You could succinctly use the start of the next sentence to end this one.*
- *“These improved conditions might include more equitable access to health care and community services, a better quality of these services, subsidized formal and informal supports, and flexible hybrid means of communication.” Which would lead to…or mean what for the (public) health of Latino persons living with ADRD and their carers? What is the overall takeaway pertaining to health or public health?*
- *The discussion and conclusions could be broadened out to medical care in general if the issues appear to also be independent of the pandemic.*

Response: We have connected the conclusion to the results and discussion and have been more specific in our statements.

##### Minor Comments

*9. Mention the United States as the target population in the abstract.*

Response: We edited the abstract to specify that it was in the United States.

*10. Typos: “The fist author also condensed” and “work in the meat packing plan industry.”*

Response: We have edited these and other typos.

*11. Clarify “To make bring rigor and validity.” Or is there a typo here?*

Response: It is a typo, which we have removed. Thank you!

*12. “To make bring rigor and validity to the research process, the interviewer used active listening techniques during the interview aimed at confirming the information shared by participants. The interviewer also emphasized the fact that participants were the experts in their experiences to reduce power differentials.” This belongs in the methods, not analysis.*

Response: We have moved this section above “Data analysis.”

*13. “[Explains how after the lockdown, the care recipient only remembers long term memories]. So, I was thinking that all the time she was locked down here because of the cold weather and COVID might have affected her more.” Avoid total paraphrasing and provide (translated) direct quotes. Or put the paraphrase as context before the quote.*

Response: We have put the paraphrasing as the context before quotes to avoid total paraphrasing.

*14. “...PCPs had to reduce physical contact with care recipients, which reduced their chance to convey warmth to their patients.” A quote here would be nice.*

Response: We added a quote for this.

*15. “This fear was not unfounded. Prior to the availability of the vaccine, caregivers and care recipients acquired COVID-19. As Latino older adults, they were at an increased risk for complications including death, causing significant chronic concern and fear.” This is useful for the introduction (and the vulnerability was discussed) and discussion but should not be a part of the results. I suggest omitting this.*

Response: We have moved that section to the discussion section.

*16. “Fourth, care recipients and PCPs highlighted the frequency and severity of depressed mood among caregivers and care recipients, especially during the lockdown due to lack of social support and social isolation. The PCPs noted that lack of social support and social isolation due to lockdown negatively impacted mood, sharing:” This is redundant. Consider condensing into 1 sentence.*

Response: We have reduced the redundancies by eliminating a sentence.

*17. “Fourth, consequences of social support were physical, psychological, and social. Examples of physical consequences include potentially reducing mortality by providing formal and informal caregiving services. Psychological consequences include clinic and family support reducing loneliness and increasing feelings of safety. Social consequences include caregivers being allowed to accompany their care recipients during clinic visits, curbside visits allowing socialization and home care services lowering isolation.” Like the barriers above this section, these feel more like they could be part of the informal or formal support codes. What are the supporting quotes? Also, it is not clear what were the consequences? What were the causes that led to the consequences?*

Response: We have restructured our themes, and these are now part of the health care and community care systems themes.

*18. “Third, consequences of the higher use of remote communication were both positive and negative.” This should just be part of the remote communication theme description.*

Response: We have restructured the themes and removed this section.

*19. “...similar to other studies, the need to rely on remote communication intensified the digital divide.” Reverse it—the digital divide was problematic given the need to rely on remote communication.*

Response: We have edited this sentence as suggested, thank you!

*20. “...for their survival” in the conclusion is a bit heavy-handed. Speak on something closer at hand in the manuscript like avoiding exposure and infection.*

### Reviewer GI [4]

#### General Comments

*This paper provides an in-depth qualitative assessment of the impact and resilience factors related to the COVID-19 pandemic among Latino families with ADRD. The authors interviewed 21 family caregivers and 23 PCPs across the United States and identified 2 primary themes that characterized the experiences of the participants, involving both the impact of the pandemic and the strategies adopted to cope with the detrimental impact of the pandemic. The topic covered is of significant importance and provides important background to better understand the impact of the COVID-19 pandemic on Latino families with ADRD, with the aim of improving the quality of care and equity among the Latino community. Overall, I think that the authors should reorganize the results to better align with the aim of the study. In my specific comments I have included specific suggestions on how to make the results more organized and succinct. To strengthen the generalizability and interpretation of the findings, the authors should also include a descriptive quantitative analysis of the interviews’ analysis, where the reader can examine the prevalence of each theme and subtheme among the 21 family members and 23 PCPs.*

#### Specific Comments

##### Major Comments

*1. The results section of the abstract is not very clear. What are the overall findings? How have the 2 themes been identified?*

Response: We have mentioned the 8 themes identified in the results section of the abstract.

*2. Introduction: Please outline the qualitative variables selected to investigate the aim of the study.*

Response: The qualitative variable selected to investigate the aim of the study was the impact of the COVID-19 pandemic. Please find this clarification in the introduction section, last paragraph.

*3. Methods:*

- *Sample and assessment: Given the qualitative nature of the study, it would be helpful if the authors included an example of a question from the interview script and also attached as an appendix the template of questions they used to direct the conversation.*
- *Data analysis: This section is not very clear and it would help if the authors could describe the process of coding in a step-by-step manner and also make the text more succinct.*

Response: We have included an appendix with the interview guides. We have restructured the methods to make the description of coding clearer.

*4. Results: The authors provide a very detailed qualitative analysis; however, more quantitative information should be provided to show the prevalence of each theme and subtheme for all the caregivers and PCPs (eg, how many PCPs reported food access and malnutrition during the COVID-19 pandemic?)*

Response: This work does not use content analysis, where quantifying the data is one of the main goals. Instead, we use thematic analysis, which focuses on using themes to generate new insights about a particular phenomenon.

##### Minor Comments

*5. In the abstract, results section, please remove the capital letters for the 2 themes and consider writing them as “ Qualitative analysis of transcripts revealed two themes: (1) the impact of a global pandemic (eg, accelerated cognitive and physical decline, or caregivers choosing between risking finances and the family’s infection given the work situation) and (2) developing resilience to the effects of the pandemic (eg, caregivers seeking vaccination sites, moving in with the care recipient and adopting telehealth.”*

Response: We have removed the capital letters from the themes and numbered them as suggested.

*6. Introduction: This sentence should be revised for clarity: “As of January of 2022, Latinos represent 8% of the US 65 and older population, but 13% of COVID-19 cases in the same age group [9].” It is not clear if the 13% of cases is the percentage of COVID-19 cases in Latino individuals aged 65 years and older.*

Response: Yes, it refers specifically to the 65 years and older population for both things: the US population and the COVID-19 cases. We have edited this sentence to say “As of January of 2022, among individuals aged 65 years and older, Latino individuals represent 8% of the US population but 13% of COVID-19 cases.”

*7. Methods:*

- *This sentence should be incorporated in the introduction or removed: “The goal of this study was to gain an in-depth understanding of the impact of the COVID-19 pandemic on Latino families with ADRD.”*
- *Who transcribed the interviews?*

Response: We deleted the sentence and edited the following sentence to express that this analysis was part of a broader study. With respect to the transcriptions, a professional team transcribed all interviews, which we mention in the methods section.

*8. Results:*

- *To improve clarity, the authors should label the descriptions of the different themes as themes (1, 2, etc) and subthemes (1.1, 1.2, 1.3, etc; 2.1, 2.2, 2.3, etc). Please add this labeling both in Table 2 and in the text below to help the reader better orient into each of the themes.*

Response: This is a very good idea. Thank you! We have added these markers.

*9. Page 6, last sentence: Please remove “make” from “To make bring rigor and validity.”*

Response: We have deleted the typo.

*10. Page 14, last paragraph: The authors should edit “work” with “works.”*

Response: We have edited this typo. Thank you!

## Round 2 Review

Dear Prof. Meinert,

Re: Manuscript ID #42211 entitled “Impact of the COVID-19 Pandemic on Latino Families With Alzheimer Disease and Related Dementias: Qualitative Interviews With Family Caregivers and Primary Care Providers.”

Enclosed is a file containing the revised manuscript by Perales-Puchalt, et al [1]. Thank you for this opportunity to resubmit the present paper.

We also thank the reviewers very much for their suggestions and comments to our manuscript. We have taken all of them into consideration for producing the new version. In the following sections, we have first presented the reviewers’ comments, followed by our responses.

We hope that these responses and the new manuscript prove satisfactory to you and your reviewers.

We are looking forward to hearing from you. Thank you again for your attention to our work.

Sincerely,

Jaime Perales-Puchalt on behalf of the authors

Fairway, KS; December 8, 2023

### Reviewer FN

#### General Comments

*The authors thoroughly attended to the reviewer responses. The methods are easy to understand and the rework of the themes and related quotes in the results is a great improvement. The discussion could be easier to read by breaking the large paragraphs into smaller ones. The conclusion should be revised to more specifically attend to what the study found and how it extends the literature. These and other issues are further noted:*

#### Specific Comments

##### Major Comments

Major comments in order of appearance in the manuscript:

*1. In the 2.2. poor nutrition theme, it is more obvious that the mailing system and financial insecurity could directly be influenced by the pandemic, but it is not clear that skills and level of impairment were affected by the pandemic. As written, it sounds more like an overarching ADRD problem rather than an ADRD issue specific to the pandemic. This should be clarified, especially since it is brought up specifically in the discussion as a unique finding.*

Response: We have clarified that the more structural issues (mailing system and financial insecurity) interacted with skills and impairment, which resulted in unhealthier options being more accessible.

*2. Consider reworking this sentence to clarify and streamline: “While home-delivered meals operated normally, Latino families with ADRD tried to access these for the first time during the pandemic to obtain food while reducing the risk of infection.” My suggested rework is “Some Latino families with ADRD we interviewed tried to use home-delivered meals for the first time during the pandemic to reduce risk of infection.”*

Response: We have replaced the sentence with the alternative provided by the reviewer. Thank you for the suggestion!

*3. Break large paragraphs throughout the discussion into smaller ones by more specific topic (eg, food and nutrition, work changes, and infection risk).*

Response: We have separated the paragraphs by topic as suggested.

*4. How exactly are fatalism and personalism related to the findings in the study? Make an explicit tie, back into the findings to make a stronger ending to this part of the discussion.*

Response: We have connected fatalism and personalism more clearly to the results from our interviews.

*5. Rephrase “Was this also the case for cognitive and functional decline?” into a statement rather than a question.*

Response: We have replaced this question with the following statement: “Studies could explore whether this disproportionate impact also applies to cognitive and functional decline.”

*6. As written, the conclusion paragraph does not indicate well what the study found or how it extends the literature. The first sentence needs to be reworked—who is “their” referring to? Families were critical to “maintaining or improving” “health and quality of life,” correct? Make succinct and specific mention to how the families were affected “beyond infection and physical symptoms.” What were the specific barriers that were exacerbated?*

Response: We have reworked the conclusions section as suggested by clarifying the ambiguous sentences and expanding on examples.

##### Minor Comments

*1. “Other caregivers or their care recipient had been infected or were indeed infected during the interview.” This sounds like the interviewer infected them. They were experiencing COVID-19 at the time of the interview?*

Response: We have edited this sentence as requested. Thank you!

*2. Typo in theme 4.3: “to the their.”*

Response: We have fixed the typo.

*3. Avoid the numeric two in the quote in theme 5.3: “Mom had 2 that got COVID.” Suggested rework: “Mom had two [home assistants] that got COVID.”*

Response: We have replaced the number with the word “two.”

*4. Remove the hyphen from “frequently-mentioned.”*

Response: We have deleted the hyphen.

### Reviewer GI

#### General Comments

*This paper provides an in-depth qualitative assessment of the impact and resilience factors related to the COVID-19 pandemic among Latino families with ADRD. The authors interviewed 21 family caregivers and 23 PCPs across the United States. They identified 2 primary themes that characterized the participants’ experiences, involving both the impact of the pandemic and the strategies adopted to cope with the detrimental impact of the pandemic. The topic covered is of significant importance and provides important background to better understand the impact of the COVID-19 pandemic on Latino families with ADRD and to improve the quality of care and equity among the Latino community. The authors did a great job improving the clarity of the methods and the results. I have included other minor revisions to further improve the clarity of the text.*

#### Specific Comments

##### Minor Comments

*1. Results*

- *Paragraph “theme 8” (line 4): Remove “and” before “healthcare”*
- *Paragraph 8.3 (line 3): Replace " requested” with “requesting.”*

Response: We have removed the word “and” and replaced “requested” with “requesting.”

*2. Discussion: Please clarify the second paragraph of the “implication and future directions” section. In the first sentence, “Our findings can inform future studies. For example, participants reported pandemic-related physical and cognitive deterioration and the importance of family support.” Can you clarify what type of findings can inform future research? Could you say something on the line of “Our findings regarding the physical and cognitive deterioration caused by the pandemic and the importance of family support may help inform future studies on…” In the same paragraph, there is a very minor typo; please replace “health” with “healthy.”*

Response: We have replaced the sentence with the one suggested by the reviewer and corrected the minor typo. Thank you!

## Abbreviations

ADRD: Alzheimer disease and related dementias
COREQ: Consolidated Criteria for Reporting Qualitative Research
PCP: primary care providers
